# Critical discussion on drug efflux in *Mycobacterium tuberculosis*

**DOI:** 10.1093/femsre/fuab050

**Published:** 2021-10-12

**Authors:** Sille Remm, Jennifer C Earp, Thomas Dick, Véronique Dartois, Markus A Seeger

**Affiliations:** Institute of Medical Microbiology, University of Zürich, Gloriastrasse 28/30, CH-8006 Zürich, Switzerland; Institute of Medical Microbiology, University of Zürich, Gloriastrasse 28/30, CH-8006 Zürich, Switzerland; Center for Discovery and Innovation, Hackensack Meridian Health, 111 Ideation Way, Nutley, NJ 07110, USA; Department of Medical Sciences, Hackensack Meridian School of Medicine, Interprofessional Health Sciences Campus, 123 Metro Boulevard, Nutley, NJ 07110, USA; Center for Discovery and Innovation, Hackensack Meridian Health, 111 Ideation Way, Nutley, NJ 07110, USA; Department of Medical Sciences, Hackensack Meridian School of Medicine, Interprofessional Health Sciences Campus, 123 Metro Boulevard, Nutley, NJ 07110, USA; Institute of Medical Microbiology, University of Zürich, Gloriastrasse 28/30, CH-8006 Zürich, Switzerland

**Keywords:** drug efflux, drug resistance, *Mycobacterium tuberculosis*, membrane transport, efflux inhibitors

## Abstract

*Mycobacterium tuberculosis* (Mtb) can withstand months of antibiotic treatment. An important goal of tuberculosis research is to shorten the treatment to reduce the burden on patients, increase adherence to the drug regimen and thereby slow down the spread of drug resistance. Inhibition of drug efflux pumps by small molecules has been advocated as a promising strategy to attack persistent Mtb and shorten therapy. Although mycobacterial drug efflux pumps have been broadly investigated, mechanistic studies are scarce. In this critical review, we shed light on drug efflux in its larger mechanistic context by considering the intricate interplay between membrane transporters annotated as drug efflux pumps, membrane energetics, efflux inhibitors and cell wall biosynthesis processes. We conclude that a great wealth of data on mycobacterial transporters is insufficient to distinguish by what mechanism they contribute to drug resistance. Recent studies suggest that some drug efflux pumps transport structural lipids of the mycobacterial cell wall and that the action of certain drug efflux inhibitors involves dissipation of the proton motive force, thereby draining the energy source of all active membrane transporters. We propose recommendations on the generation and interpretation of drug efflux data to reduce ambiguities and promote assigning novel roles to mycobacterial membrane transporters.

## Teaser

This review provides an overview of mycobacterial drug efflux pumps and sets out recommendations on how to perform and interpret drug efflux experiments.

## INTRODUCTION

The threat of tuberculosis, a disease caused by *Mycobacterium tuberculosis* (Mtb), to public health cannot be overstated. In recent years, it has been the only infectious disease caused by a single agent in the top 10 list of global causes of death. Every year, ∼10 million people fall ill with tuberculosis and in 2019, 1.4 million tuberculosis patients died (World Health Organization 2020). The predicted impact of the COVID-19 pandemic is an additional 190 000 TB deaths in 2020 and up to 20% increase of the global disease burden in the next 5 years (Alene, Wangdi and Clements 2020; Glaziou 2020; Hogan *et al*. 2020). Treatment of uncomplicated drug-susceptible tuberculosis includes four antibiotics taken daily for 2 months followed by two antibiotics taken for an additional 4 months, with frequent side effects. Additionally, drug-resistant cases of Mtb, which require more extensive treatment with second-line antibiotics, are increasing. In 2019, rifampicin-resistant and multidrug-resistant tuberculosis cases accounted for 4.6% of all tuberculosis cases (World Health Organization 2020).

Mtb exploits different intrinsic mechanisms to resist drug therapy. One of the most important defence features of this pathogen is its complex, hydrophobic cell envelope that prevents the influx of many drugs (Sarathy, Dartois and Lee 2012). For drugs that can access their targets, mutations that alter the target (for example, *rpoB* mutations in rifampicin resistance; Zaw, Emran and Lin 2018) or prevent prodrug activation (for example, *katG* mutations in isoniazid resistance; Vilchèze and Jacobs 2014) have arisen, rendering the compounds ineffective. Another mechanism by which Mtb could withstand antibiotic treatment is drug efflux.

Many reviews were written over the past two decades describing drug efflux in mycobacteria (Sarathy, Dartois and Lee 2012; Black *et al*. 2014; te Brake *et al*. 2018; Kanji, Hasan and Hasan 2019). Here, we approach the topic from a new angle. In recent years, we and others have published results that have important implications for the interpretation of research conducted on drug efflux. We hope to draw attention to the ambiguities we have encountered and to critically analyse the conclusions drawn about the functions of putative drug efflux pumps.

A topic inherently related to drug efflux pumps is their inhibition by efflux inhibitors (EIs), which are studied extensively due to their clinical potential as adjunctive therapy. In the literature, the more specific term ‘efflux pump inhibitors’ is used often and liberally (Bhardwaj and Mohanty 2012). The term in its strictest sense means compounds that actively bind efflux pumps and thus obstruct the transport of their substrates. However, in many studies compounds that dissipate the energy source of the transporter, be it proton motive force (PMF) or ATP, are also described as ‘efflux pump inhibitors’, even though the term in a more general manner as ‘efflux inhibitor’ may be more appropriate. In this review, we summarize the current knowledge on the modes of action of efflux inhibitors most commonly used in studies characterizing efflux pumps in mycobacteria.

The main goal of our review is to assess transporters proposed to act as drug efflux pumps. To this end, we reevaluate experimental data from original publications and interpret these initial findings in the context of more recent studies.

## EFFLUX INHIBITORS

**Figure 1.**
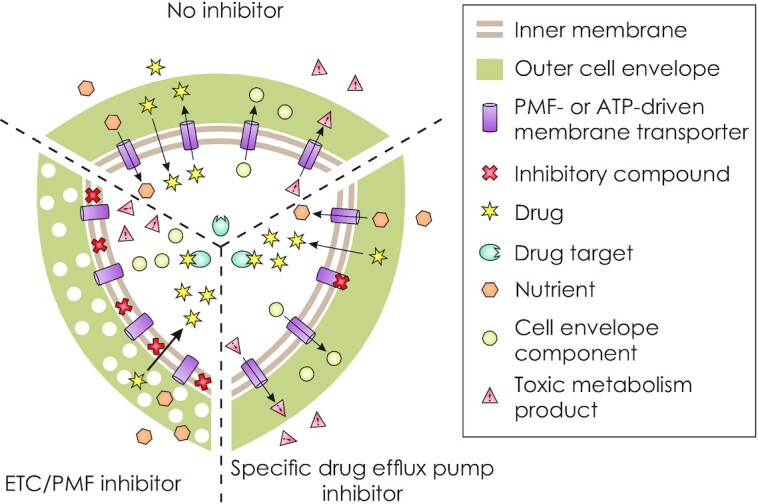
Transport of nutrients (hexagons), drugs (stars), cell envelope components (spheres) and toxic metabolites (triangles) across mycobacterial inner membrane by PMF or ATP-driven transport proteins (cylinders). When no inhibitor is present, all transport processes follow their natural course and the mycobacterial cell thrives. The drug is pumped out of the cell by a drug efflux pump before it reaches its target (light blue oval). If an efflux pump inhibitor blocks drug export, the drug can reach its target and inhibit growth or kill the cell, although other transporters still fulfil their functions. If, however, the inhibitory compound affects component(s) of the electron transfer chain (ETC) or dissipates the PMF, all active membrane transporters are suppressed, resulting in broader metabolic damage to the cell. Further, drugs might gain better access to the cytosol and their targets due to a more permeable cell envelope.

EIs are invaluable to drug efflux pump research and drug discovery. Some EIs have been proposed for clinical use (Srikrishna *et al*. 2015; Amaral and Viveiros 2017)—to inhibit efflux pumps and keep the drugs within the cells, enabling them to act on their targets. It is important to distinguish compounds that are truly efflux pump inhibitors and bind directly to transporters, from molecules that potentiate the effect of antibiotics in bacteria in other ways, for example by cutting off the energy source (PMF or ATP) from all transport proteins (Fig. 1). In the latter case, not only drug efflux pumps but also import of nutrients and export of cell envelope components and toxic metabolism products (Jones, Hernández Lozada and Pfleger 2015) are disrupted. These processes are essential for bacterial survival and their interruption leads to a general decrease of viability.

In this section, we have summarized current knowledge on the mode of action of efflux inhibitors, many of which have been widely regarded as direct efflux pump inhibitors in the mycobacterial research field and in microbiology in general.

## CCCP

**Figure 2.**
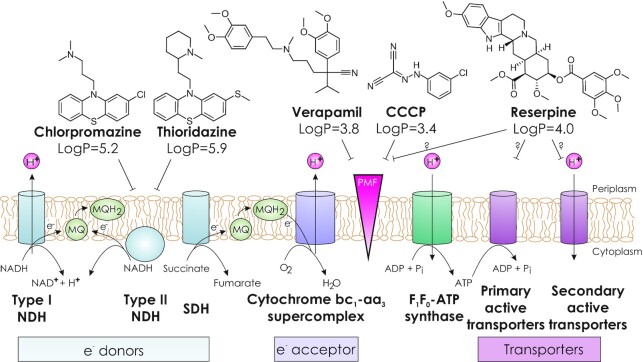
Interplay between oxidative phosphorylation (under aerobic conditions), membrane transporters and inhibitory compounds CCCP, chlorpromazine, reserpine, thioridazine (TDZ) and verapamil in Mtb. Lack of proton gradient across the inner membrane, either by dissipation or inhibition of its formation (via blocking the ETC), results in inactivation of both primary and secondary active transporters, which harness ATP hydrolysis or proton translocation as an energy source, respectively. The LogP values (calculated by XLogP3 software) are octanol–water partition coefficients that illustrate the hydrophobicity of the inhibitory compounds that promotes insertion into membranes. NDH—NADH dehydrogenase, SDH—succinate:menaquinone oxidoreductase, PMF—proton motive force. Refer to Black *et al*. (2014) and Cook *et al*. (2014) reviews for further information about ETC and oxidative phosphorylation in mycobacteria.

Carbonyl cyanide *m*-chlorophenylhydrazone (CCCP) is a protonophore, known to dissipate the chemical component of PMF by equilibrating proton concentrations on both sides of the membrane (Plášek, Babuka and Hoefer 2017). As a consequence of CCCP treatment, the energy source of all membrane transporters is disrupted. The PMF is rapidly abolished, eliminating the energy source of all secondary (PMF-dependent) active transporters. Eventually, the primary active ABC (ATP-binding cassette) transporters are also affected, due to the loss of proton gradient-dependent F_1_F _0_-ATP synthase activity (Fig. 2) (Black *et al*. 2014; Cook *et al*. 2014). The deprivation of energy affects all transport processes, including export of cell envelope components and import of nutrients.

### Phenothiazines

Phenothiazines, such as chlorpromazine and thioridazine (TDZ), are heterocyclic compounds that have a long history of use in psychiatric diseases. The compounds also have antimicrobial and efflux inhibitory properties that were reviewed recently by Grimsey and Piddock (2019). Their ability to potentiate effects of antibiotics against pathogens such as Mtb and methicillin-resistant *Staphylococcus aureus* have generated interest in the tuberculosis field (Adams, Szumowski and Ramakrishnan 2014; Amaral and Viveiros 2017). Detailed biochemical studies showed that in Mtb, phenothiazines specifically inhibit type II NADH dehydrogenases (both Ndh and NdhA in Mtb) that transfer electrons from NADH to menaquinone in the respiratory chain, thus obstructing oxidative phosphorylation (Fig. 2) (Boshoff *et al*. 2004; Weinstein *et al*. 2005). Inhibition of the respiratory chain is supported by the findings that a 3 h incubation with TDZ significantly reduces the NADH/NAD^+^ ratio in Mtb compared with an untreated control (Dutta, Mehra and Kaushal 2010). After one day of exposure to TDZ and chlorpromazine, the intracellular ATP levels in Mtb are drastically diminished (Machado *et al*. 2016). Since the respiratory chain is disrupted, both PMF and ATP are depleted, which impacts the export of native substrates and drugs by all classes of transporters. In fact, transcriptome (Dutta, Mehra and Kaushal 2010) and proteome (De Keijzer *et al*. 2016) studies of Mtb treated with TDZ have suggested modulation of expression and production of many proteins involved in lipid metabolism, cell wall processes, and intermediary metabolism and respiration, thereby leading to increased permeability of the cell envelope and altering lipid composition of the plasma membrane (De Keijzer *et al*. 2016).

However, some experiments suggest that phenothiazines inhibit drug efflux by interacting directly with transporters. Te Brake *et al*. used inside-out membrane vesicles from HEK293 cells where selected human multidrug ABC transporters were overexpressed to evaluate the inhibitory effect of anti-tuberculosis compounds on these transporters. TDZ was shown to inhibit the transport of radioactively labelled model substrates ([^3^H]N-methyl quinidine and [^3^H]estrone sulfate) by ABCB1 (P-glycoprotein) and BCRP when ATP was added externally (te Brake *et al*. 2016). Phenothiazines have also been implicated as substrates and inhibitors of AcrB efflux pump both in *Escherichia coli* and *Salmonella enterica* serovar Typhimurium (Bailey, Paulsen and Piddock 2008).

Unfortunately, similar experiments have not been done with mycobacterial transporters; whether inhibition of chlorpromazine and TDZ is caused by an indirect effect on membrane energetics, by direct binding to the efflux pumps or a combination of both, remains to be determined.

### Reserpine

Reserpine is an alkaloid found in the roots of *Rauvolfia serpentina* and *Rauvolfia vomitoria* and was approved as a drug in the 1950s to treat patients suffering from hypertension and psychotic symptoms. In the human body, reserpine strongly binds to and inhibits the vesicular monoamine transporter 2 (VMAT2, SLC18A2) found in chromaffin granule membranes where it imports biogenic amines such as serotonin, dopamine, epinephrine and norepinephrine (Rudnick *et al*. 1990; Stern-Bach *et al*. 1990; Sievert, Hajipour and Ruoho 2007). In experiments with membrane vesicles from two different human cancerous cell lines overexpressing ABCB1 (P-glycoprotein, an ABC transporter responsible for multidrug resistance in humans) and displaying a multidrug resistance phenotype, reserpine was able to inhibit photolabeling of ABCB1 by a radioactively labelled vinblastine (chemotherapy compound) analogue [^125^I]NASV, suggesting direct binding of reserpine to ABCB1 (Akiyama *et al*. 1988; Beck *et al*. 1988).

In bacteria, reserpine was described as a drug efflux pump inhibitor, mainly in the context of major facilitator superfamily (MFS) transporters of gram-positive bacteria such as NorA of *S. aureus* (Ng, Trucksis and Hooper 1994; Schmitz *et al*. 1998; Holler *et al*. 2012) and Bmr of *Bacillus subtilis* (Ahmed *et al*. 1993), but also MdfA of *E. coli* (Edgar and Bibi 1999; Liu *et al*. 2016a). An elegant mutational study on Bmr, a multidrug transporter from *B. subtilis*, found that mutations of two phenylalanine residues (F143 and F306) reduced the inhibition of Bmr by reserpine (Klyachko, Schuldiner and Neyfakh 1997). In addition, these mutations also altered the substrate specificity of Bmr, differentially affecting transport of various drugs. This suggests that reserpine shares its binding site with other drugs and argues against an indirect mechanism of inhibition (Klyachko, Schuldiner and Neyfakh 1997).

**Figure 3.**
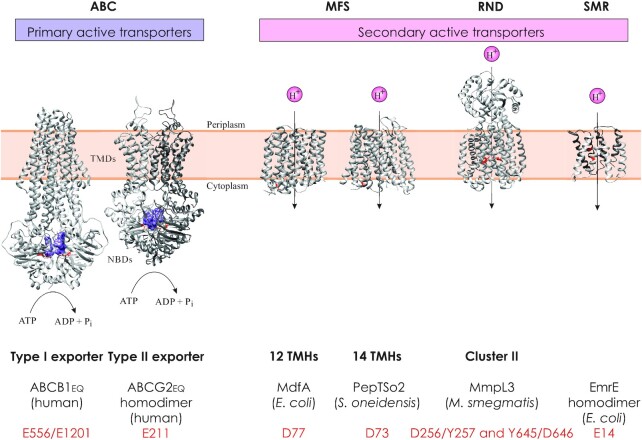
Representative structures of transporters of the ABC, MFS, resistance-nodulation and cell division (RND) and small multidrug resistance (SMR) superfamilies (when available, from mycobacteria) and their conserved catalytic residues. The monomeric units of dimers are coloured in different shades of grey and the conserved catalytic residues of the representative structures are highlighted in red. Primary active transporters: type I and type II exporters of the ABC superfamily in an outward-open conformation with dimerized nucleotide binding domains (NBDs) that are bound to ATP (purple surface) and transmembrane domains (TMDs) that are open towards the periplasm. The two types differ in the length and organization of their TMDs. Secondary active transporters: The MFS can be subdivided into subfamilies with 12 and 14 transmembrane helices (TMHs). Both types can be inactivated via mutation of a conserved aspartic acid residue located in motif A. The mycobacterial membrane protein large (MmpL) transporter family belongs to the RND superfamily. Based on topology predictions, it can be subdivided into two clusters. Structural information is only available for cluster II proteins, but sequence alignments suggest that the catalytic residues are conserved between the two clusters (Bernut *et al*.^2016^). PDB identifiers: ABCB1 (6C0V), ABCG2 (6HBU), MdfA (4ZOW), PepT_So2_ (4LEP), MmpL3 (6AJF)—shown without its flexible C-terminal domain, EmrE (3B5D). See the review by Du *et al*. (2018) for further information on the structures of different superfamilies.

Curiously, the transporters that reserpine has been shown to bind belong to different families (Fig. 3). While ABCB1 is an ABC transporter, VMAT2, NorA, MdfA and Bmr are all MFS transporters. It would be interesting to determine how reserpine is able to bind transporters with such different structures (Fig. 3) and whether the requisites for reserpine binding are similar in different classes of transporters.

In mycobacteria, direct inhibition of drug efflux pumps by reserpine has not been formally shown (Louw *et al*. 2011). In one study, a small and statistically nonsignificant, but reproducible increase of rifampicin accumulation was detected in different mycobacterial species upon treatment with reserpine (Piddock, Williams and Ricci 2000). In the same study, however, addition of glucose abolished this effect in *M. smegmatis*(Msm) and *M. aurum*, suggesting reserpine inhibition can be reversed by energization of the bacterial cells (Piddock, Williams and Ricci 2000). Similar experiments with glycerol and reserpine have been carried out in the study of the RND transporter MmpL7 and the ABC transporter Rv2686c-2688c (Pasca *et al*. 2004, 2005). Thus, circumstantial evidence exists that reserpine could also affect the energetics of mycobacteria. To our knowledge, the possibility of reserpine acting as an uncoupling agent has only been investigated in mammalian mitochondria (Maina 1974; Weinbach *et al*. 1983).

### Verapamil

Verapamil was approved in the 1980s for the treatment of several cardiovascular diseases because of its inhibitory activity of voltage-dependent Ca^2+^ channels. A crystal structure of a homotetrameric bacterial Ca^2+^channel revealed that verapamil blocks the Ca^2+^ selectivity filter from the intracellular side (Tang *et al*. 2016). Like reserpine, verapamil has been implicated in inhibition of ABCB1. This was initially proposed by Shapiro and Ling in 1995, when they showed reduced export of Hoechst 33342 by ABCB1 in reconstituted liposomes upon treatment with verapamil (Shapiro and Ling 1995). However, several decades of drug efflux inhibitor research on ABCB1 has brought about much stronger and highly specific inhibitors of ABCB1, such as zosuquidar for which a cryo-EM structure was recently solved (Alam *et al*. 2019). Therefore, the current view in the ABCB1 field is that verapamil is a weak inhibitor (Robey *et al*. 2008) or even an ABCB1 substrate, because it was used as such in a recent study (Bauer *et al*. 2017).

Ramakrishnan and colleagues have suggested that verapamil is an inhibitor of drug efflux pumps in mycobacteria (Adams *et al*. 2011; Adams, Szumowski and Ramakrishnan 2014). It was shown that replicating populations of Mtb and *M. marinum* develop drug tolerance upon residence in macrophages (Adams *et al*. 2011). They suggest that this phenomenon is due to induction of drug efflux pumps of Mtb residing in macrophages, since transposon insertion in the Tap (Rv1258c) transporter gene reduces rifampicin tolerance compared with the wild-type (WT) strain (Adams *et al*. 2011). Verapamil was shown to decrease macrophage-induced tolerance of some drugs (Adams *et al*. 2011; Adams, Szumowski and Ramakrishnan 2014). Interestingly, the major verapamil metabolite norverapamil as well as the R-enantiomer of verapamil show similar reduction of drug tolerance, but have strongly reduced cardiac activity, providing an opportunity for higher dosing in humans (Adams, Szumowski and Ramakrishnan 2014). In mice, addition of verapamil to the standard tuberculosis treatment showed promise in shortening first-line therapy duration and reducing relapse rates, especially in the C3HeB/FeJ mouse model that develops necrotic lesions with many extracellular bacilli exposed to hypoxic conditions (Gupta *et al*. 2013).

The prevailing opinion on the mechanism of action of verapamil is direct inhibition of mycobacterial drug efflux pumps (Adams *et al*. 2011; Adams, Szumowski and Ramakrishnan 2014). However, a recent study by Dartois and colleagues showed that intracellular accumulation of bedaquiline, clofazimine, rifampin, isoniazid, pyrazinamide, moxifloxacin, linezolid and ethambutol was not affected by pretreatment of Mtb with verapamil (Chen *et al*. 2018). Instead, verapamil was shown to impair the ΔpH component of the PMF (Chen *et al*. 2018). Similarly, an elegant study focusing on the subcellular localization of bedaquiline in macrophages showed that addition of verapamil to macrophages infected with Mtb did not increase the intrabacterial concentration of bedaquiline (Greenwood *et al*. 2019). Although the permeability of cell membranes does not seem to be compromised upon verapamil treatment, neither in the short term nor in the long term (Chen *et al*. 2018), the morphology of the cells is already affected after 16 h, suggesting an effect on the cell envelope (Caleffi-Ferracioli *et al*. 2016). This observation is consistent with the amphiphilic nature of verapamil, favouring nonspecific insertion into lipid bilayers (Meier *et al*. 2006). Together, these findings suggest a more indirect role for verapamil in the inhibition of transport processes in mycobacteria, rather than direct inhibition of drug efflux pumps (Fig. 2). Likewise, enhanced efficacy of the first-line regimen (Gupta *et al*. 2013) and bedaquiline (Xu *et al*. 2018) by adjunctive verapamil is likely due to improved systemic exposure to rifampicin and bedaquiline, respectively, via inhibition of mammalian transporters, not bacterial efflux pumps (Chen *et al*. 2018; Xu *et al*. 2018). Importantly, these findings do not invalidate the observations by Ramakrishnan and colleagues that verapamil dampens drug tolerance in Mtb. However, it appears unlikely that drug efflux itself plays a key role in the process. Rather, it is plausible that membrane transporters annotated as drug efflux pumps contribute to this phenomenon via mechanisms that await to be discovered.

## IDENTIFICATION OF TRANSPORTERS AS DRUG EFFLUX PUMPS: DOS AND DON'TS

### Recommendations on how to characterize drug efflux pumps

Drug efflux pumps are found within five membrane transporter superfamilies, namely the ABC, RND, MFS, MATE (multidrug and toxic compound extrusion) and SMR transporters (Fig. 3) (Du *et al*. 2018). Many transporters of these five superfamilies are annotated as drug efflux pumps on the basis of sequence conservation with characterized drug transporters. While such annotations provide a first hint of whether a membrane protein of interest could be involved in drug efflux, a recent study on bacterial ABC transporters revealed that such predictions based on sequence homology can be misleading (Hürlimann *et al*. 2016), highlighting the importance of investigating predicted functions experimentally.

The classical starting experiment is to delete the gene of a presumed drug efflux pump, followed by the determination of minimal inhibitory concentrations (MICs) of antibiotics in comparison to the WT strain. Provided one observes a reduction in MIC, the investigated transporter could be a drug efflux pump. However, such a result cannot be interpreted unambiguously: (i) the sheer presence or absence of a transporter protein may lead to a fitness difference, which may not manifest itself as a growth defect in the absence of drugs, but only in the presence of drug-induced stress; (ii) if the transporter gene is part of an operon, its deletion may impact the expression of additional proteins; and (iii) the deleted transporter may in fact be responsible for the biogenesis of bacterial membranes or cell envelope and thereby the deletion might lead to a reduced barrier function and increased drug influx.

It is important to note that the genomic deletion of a drug efflux pump does not necessarily need to manifest in an MIC difference, because the loss might be compensated by the upregulation of other drug efflux pumps with redundant substrate spectrum (Blair *et al*. 2015) or the investigated drug efflux pump may not be expressed at high enough levels in the WT cells to confer drug resistance (Hürlimann *et al*. 2016). For this reason, heterologous overexpression of the candidate drug efflux pump is frequently applied to provide complementary genetic evidence supporting drug efflux activity.

Once gene deletion or overexpression experiments provided first evidence for drug efflux activity, an in-depth characterization should be carried out.

- In case a gene deletion showed a drug susceptibility phenotype, the deleted gene needs to be complemented by expressing the transporter *in trans*. Drug efflux pumps are active transporters that contain highly conserved residues to couple substrate transport to the translocation of protons (in case of secondary-active transporters) or the hydrolysis of ATP (in case of primary-active ABC transporters) (Fig. 3). These residues should be mutated to inactivate the transporter function and such inactivated transporter mutants should be included in functional experiments. Sequence alignments and homology modelling are useful tools to identify the conserved residues in the absence of a structure (Fig. 3), which is the case for the majority of mycobacterial transporters. As a side note, the introduction of a single inactivating transporter mutation directly at the level of the genome (instead of the gene deletion) represents the most precise procedure to study a drug efflux pump in its native context, but for practical reasons is rarely chosen.
- Ideally, heterologous expression of the transporter of interest in another organism is performed to determine whether resistance to the antibiotics in question is induced. In these experiments, it is paramount to compare WT transporter and its inactive mutant side by side. Often, genes encoding dominant native drug efflux pumps must be deleted in the host organism chosen for heterologous expression (for example, the tripartite *acrAB-tolC* system in *E. coli* [Ma *et al*. 1995] or the heterodimeric ABC transporter *lmrCD* in *Lactococcus lactis* [Lubelski *et al*. 2006]) to enable meaningful result interpretation.
- Side-by-side MIC and transport assays using radioactively labelled drugs or fluorescent substrates such as ethidium bromide, BCECF-AM or Hoechst 33342, with inactive transporter mutants as control, can help establish a causal effect (Blair and Piddock 2016). Transport assays are often performed in bacterial cells and cell-derived membrane vesicles. One must keep in mind that in the experimental systems of intact cells and membrane vesicles all components of the native membranes are present and the effects of antibiotics and EIs might be indirect, especially in live cells. The most adequate transport assays would involve purification and reconstitution of the transporter in proteoliposomes. Using this strategy, all components necessary for drug transport can be systematically tested and the nature of the transporter as a drug efflux pump can be established unambiguously. However, such transport assays are difficult to perform because drugs and dyes recognized by drug efflux pumps are typically hydrophobic and therefore interact nonspecifically with membranes. As a cautionary note, fluorescent dyes can be pH-sensitive and prone to self-quenching, as was described in detail for Hoechst 33342 (Verchère, Broutin and Picard 2013).
- We consider it important to choose an experimental set-up that allows to distinguish between drug efflux directly mediated by the investigated transporter and drug influx, which can be indirectly influenced by the investigated transporter. Such distinction was recently established for the mycobacterial MFS transporter Rv1410c (Hohl *et al*. 2019) (see below). Short incubations with proton uncouplers to quickly remove the energy source for the investigated transporter play a key role in such experiments.
- Ideally, structural biology approaches such as X-ray crystallography or single particle cryo-EM are employed to solve structures of the investigated transporter binding the drug to which it is supposed to confer resistance. Unfortunately, the resolution of the structures or the occupancy of the drug binding site are often insufficient to obtain unambiguous drug binding data (Liu *et al*. 2016a). In addition, structural studies of membrane proteins are notoriously challenging, need specialized infrastructure and thus can only be expected for selected transporter–drug combinations.

### Common pitfalls

In reviewing the literature, we have identified three potential pitfalls that can lead to misinterpretation of drug efflux data.

- It is common practice to determine MIC values in the presence and absence of efflux inhibitors. The data from such MIC assays need cautious interpretation as the timescale of the experiment usually spans many bacterial replication cycles, allowing for other processes to affect antibiotic action. For example, a transporter might shuttle cell envelope components to the periplasm and its absence or reduced activity might result in increased permeability to the antibiotics (Fig. 1). Some efflux inhibitors such as CCCP, TDZ and verapamil have a broader influence on the metabolism of mycobacteria than direct binding and inhibition of transporters (Fig. 2). Thus, given enough time, PMF, oxidative phosphorylation or membrane potential is disrupted by these compounds and many cellular (transport) pathways are affected.
- In many studies, the upregulation of drug efflux pumps at the transcript or protein level is considered proof that the cell's drug efflux machinery was switched on. However, overexpression of transporters might simply be a cellular response to compensate for stress-induced changes in the metabolism or cell envelope by importing or exporting substances other than drugs.
- In experiments in which a drug efflux pump is expressed in a heterologous host, the phenotype is often compared to cells bearing an empty vector control. However, the results from such experiments might be misleading because the overexpression of a transporter is a burden to the cell and the empty vector control can also differentially affect cell growth. Even in the absence of drugs, we often observe growth differences between cells expressing the transporter versus cells harbouring the empty vector. For this reason, it is important to include an inactivated transporter mutant as the control.

## DRUG EFFLUX IN *M. TUBERCULOSIS*

In the following section, we present an overview of selected Mtb transporters that have been described in the literature as contributing to drug efflux. We discuss the data on which these claims are based (summarized in Table S1, Supporting Information) and what conclusions can be drawn. The transporters are ordered according to the strength of experimental evidence supporting a proposed function as efflux pump, in decreasing order. This is followed by a summary of the putative drug efflux pumps that were attributed other functions after in-depth characterization (which does not exclude an additional function in drug efflux, but is unlikely in some of the outlined cases).

### (Putative) Drug efflux pumps in Mtb

#### Rv0676c (MmpL5)

The RND transporter mycobacterial membrane protein large (MmpL) 5 and its periplasmic accessory protein, the mycobacterial membrane protein small (MmpS) 5, form a redundant system with the MmpL4-MmpS4 proteins that export siderophores called mycobactins for iron scavenging, and are upregulated under iron starvation (Rodriguez *et al*. 2002; Wells *et al*. 2013; Jones *et al*. 2014; Zhang *et al*. 2020). Additionally, upregulation of the MmpL5-MmpS5 complex has been linked to increased resistance to bedaquiline, clofazimine (Andries *et al*. 2014; Hartkoorn, Uplekar and Cole 2014) and azoles (Milano *et al*. 2009) in Mtb, suggesting that it is a drug efflux pump. Analogous observations were reported for *M. bovis* (Milano *et al*. 2009),*M. abscessus* (Halloum *et al*. 2017; Li *et al*. 2018; Richard *et al*. 2019), *M. intracellulare* (Alexander *et al*. 2017) and *M*. *smegmatis* (Msm) (Maslov *et al*. 2020). Upregulation is caused by mutations in the transcriptional repressor of the MmpL5-MmpS5 operon, called Rv0678c in Mtb, which were found in spontaneous resistance mutants *in vitro* (Milano *et al*. 2009; Hartkoorn, Uplekar and Cole 2014; Richard *et al*. 2019; Maslov *et al*. 2020) as well as in clinical isolates (Andries *et al*. 2014; Alexander *et al*. 2017; Pang *et al*. 2017; Li *et al*. 2018) resistant to bedaquiline. Bedaquiline, a TB drug of the latest generation, and its associated resistance mechanisms, involving either target modification in the ATP synthase gene *atpE* and/or the frequently observed mutations in Rv0678c, are of high clinical importance. Mutations in Rv0678c and the resulting up-regulation of MmpL5-MmpS5 in Mtb are to the best of our knowledge the only example in which a mycobacterial drug efflux pump contributes to drug resistance in clinical settings.

Besides Mtb, mutations in the *M. abscessus* Rv0678c homologue MAB_4384 correlated with increased MICs of thiacetazone derivatives (Halloum *et al*. 2017) and bedaquiline (Li *et al*. 2018), and strains with mutations in MAB_2299c, another Rv0678c homologue, have increased clofazimine and bedaquiline MICs (Richard *et al*. 2019). In the same study, it was shown that genetic deletion of the *mmpL-mmpS* operon within this *M. abscessus* Δ*MAB_2299c* strain restored clofazimine susceptibility to that of the WT (Richard *et al*. 2019), providing a causal relationship between MmpL5-MmpS5 and drug resistance.

Given its clinical importance, surprisingly little is known on the molecular mechanism of the MmpL5-MmpS5 operon. Overexpression studies of MmpL5 and MmpS5, alone and as an operon, suggest that both operon members are needed to confer bedaquiline and clofazimine resistance (Andries *et al*. 2014). An important question is whether bedaquiline is directly transported by MmpL5-MmpS5. To this end, transport assays using [^3^H]econazole were carried out in a *M. bovis* WT strain and a strain carrying a mutation in the Rv0678c homologue. The mutant accumulated [^3^H]econazole much more slowly than WT, but rapidly reached WT accumulation levels upon addition of CCCP (Milano *et al*. 2009). These experiments clearly suggest the active efflux of azoles mediated by MmpL5-MmpS5 harnessing the PMF as an energy source.

#### Rv1258c (Tap)

Tap, or Rv1258c in Mtb, is an MFS transporter. Tap and its homologue Tap_for_ from *Mycobacterium fortuitum* have been extensively studied in Msm (Aínsa *et al*. 1998; Ramón-García *et al*. 2006), *M. bovis* BCG (Ramón-García *et al*. 2012) and Mtb H37Ra (Liu *et al*. 2019). When *rv1258c* was expressed in Msm, MIC of tetracycline increased 4-fold, compared with the empty vector control, but not to other drugs tested (Aínsa *et al*. 1998). However, when *rv1258c* was expressed from another vector in Msm, increased resistance was detected to streptomycin, gentamicin, 2′-*N*-ethylnetilmicin and 6′-*N*-ethylnetilmicin in addition to tetracycline (De Rossi *et al*. 2002). Two studies in which *tap* was either deleted in *M. bovis* BCG (Ramón-García *et al*. 2012) or Mtb H37Rv (Balganesh *et al*. 2012) added spectinomycin, gentamicin, acriflavine and aminosalicylic acid to the list of suggested effluxed substrates. Whole cell transport experiments using Msm overexpressing *tap_for_* provided direct evidence that the transporter effluxes [^3^H]tetracycline and that transport is inhibited by the addition of CCCP (Ramón-García *et al*. 2006). Interestingly, transposon-insertion mutants of Rv1258c in Mtb do not develop tolerance to rifampicin after incubation in macrophages, in contrast to the WT strain (Adams *et al*. 2011). Lee *et al*. generated semi-synthetic spectinomycin analogues that were no longer susceptible to Rv1258c-mediated drug efflux in Mtb, lending further support to the potential clinical relevance of this efflux pump (Lee *et al*. 2014).

Liu and colleagues introduced mutations detected in the *tap* gene in drug-resistant clinical isolates of Mtb to the genome of avirulent Mtb strain H37Ra, resulting in decreased sensitivity to isoniazid, pyrazinamide and streptomycin (Liu *et al*. 2019). Indeed, [^14^C]pyrazinamide/[^14^C]pyrazinoic acid accumulation in intact Mtb cells was markedly reduced in the mutant strains. However, an experimental caveat was the lack of a negative control that would demonstrate the loss of efflux when PMF is abolished (Liu *et al*. 2019).

Tap gene overexpression in Mtb has been detected in response to treatment with isoniazid (Gupta *et al*. 2006; Machado *et al*. 2012), rifampicin (Siddiqi *et al*. 2004; Gupta *et al*. 2006; Sharma *et al*. 2010), ofloxacin (Siddiqi *et al*. 2004; Gupta *et al*. 2006), ethambutol (Gupta *et al*. 2006) and streptomycin (Gupta *et al*. 2006). However, Rv1258c has not been shown to confer resistance to isoniazid and rifampicin (Ramón-García *et al*. 2012), thus calling the relevance of such drug efflux gene expression studies into question.

The *tap* deletion strain of *M. bovis* BCG was also more sensitive to vancomycin (Ramón-García *et al*. 2012). As vancomycin's target is located in the periplasm, this hints at increased drug influx mediated by lack of this transporter. Interestingly, the knock-out (KO) strain exhibited slower growth and earlier growth arrest than the WT strain in liquid media. The cell morphology was also affected, as the KO cells were elongated compared with WT cells, while gene expression was altered for the KO strain in stationary growth phase (Ramón-García *et al*. 2012). Hence, Rv1258c has a physiological role beyond drug efflux and has been hypothesized to act as an exporter of toxic compounds that would otherwise accumulate in the cells during stationary phase (Ramón-García *et al*. 2012).

#### Rv3065 (Mmr)

Mmr (Rv3065, EmrE) belongs to the family of SMR transporters. When Mtb *mmr* was expressed in Msm, it conferred resistance to tetraphenylphosphonium (TPP), ethidium bromide, erythromycin, acriflavine, safranin O and pyronin Y, as determined by at least 4-fold increased MIC values, compared with a control harbouring only the cloning vector. Incubation with CCCP restored baseline MIC values for these compounds (De Rossi *et al*. 1998). Msm producing the Mmr transporter accumulated 4.5-fold less [^3^H]TPP than the empty vector control strain and upon addition of CCCP, [^3^H]TPP accumulation levels were similar in both strains (De Rossi *et al*. 1998). Increased susceptibility towards dyes (TPP and ethidium) but not TB drugs was later observed in an *mmr* KO of Mtb, while overexpression of the pump had a modest effect on dye efflux (Rodrigues *et al*. 2013). Ethidium transport assays in intact Mtb cells (WT and gene deletion) suggest Mmr-mediated dye efflux, which can be inhibited by verapamil, chlorpromazine or CCCP (Rodrigues *et al*. 2013).

In conclusion, Mmr is capable of dye efflux, but does not play a role in the transport of clinically relevant TB drugs.

#### Rv2686c-2688c

The ABC transporter Rv2686c-Rv2688c is composed of the nucleotide binding domain Rv2686c and the two transmembrane components Rv2687c and Rv2688c, which form a heteromeric complex (Rv2686c_2_/Rv2687c/Rv2688c) with the fold of type II ABC exporters (Fig. 3) (Rempel, Stanek and Slotboom 2019). Overexpression of the Mtb *rv2686c-rv2688c* operon from plasmids in Msm decreased the sensitivity to ciprofloxacin and norfloxacin compared with vector control, demonstrated by an 8-fold and 2-fold increase in MIC, respectively (Pasca *et al*. 2004). The MIC of ciprofloxacin was reduced by addition of reserpine, CCCP or verapamil. Employing the natural fluorescence of fluoroquinolones, it was shown that ciprofloxacin accumulation was reduced by 60% compared with a vector control when Rv2686c-Rv2688c was expressed, but increased upon the addition of reserpine (Pasca *et al*. 2004). When glycerol was added to the reserpine treated sample to investigate the effect of replenishment of the energy source, a rapid decrease of ciprofloxacin levels was detected. These data strongly suggest that Rv2686c-Rv2688c exports ciprofloxacin via an energy dependent process. Interestingly, mutations in Rv2688c were also associated with fluoroquinolone resistance in a genome-wide association study of 6465 Mtb clinical isolates (Coll *et al*. 2018).

Drug efflux by Rv2686c-2688c has not been further characterized since the initial study in 2004 (Pasca *et al*. 2004), which lacked an inactivated mutant control. Additionally, the *rv2686c-rv2688c* operon has not been deleted in Mtb, but deletion of the corresponding Msm homologue MSMEG_1502–1504, which shares 70% sequence identity with Rv2686c-Rv2688c, did not have an impact on drug resistance, including the fluoroquinolones ofloxacin and ciprofloxacin (Arnold *et al*. 2018). This could be due to a compensatory effect by other Msm transporters implicated in fluoroquinolone efflux, such as the MFS transporter LfrA (Liu, Takiff and Nikaido 1996).

#### Rv0194

Rv0194 is an ABC transporter upregulated under hypoxic conditions (Liu *et al*. 2016b). The *M. bovis* homologue of this putative drug efflux pump, Bcg0231 (Mb0200), was identified in a transposon screen performed to discover proteins conferring β-lactam resistance in mycobacteria. Expression of Bcg0231 was upregulated by a transposon insertion 54 bp upstream of the coding sequence, which correlated with a 32- to 64-fold increase of the MICs of this transposon mutant for ampicillin, streptomycin and chloramphenicol, and a 4- to 8-fold increase of the MICs of vancomycin and tetracycline (Danilchanka, Mailaender and Niederweis 2008). The MICs of ampicillin, vancomycin, novobiocin and erythromycin also increased when Rv0194 was expressed from a plasmid in Msm (Danilchanka, Mailaender and Niederweis 2008). An accumulation assay with ethidium bromide showed reduced ethidium accumulation, which increased upon addition of reserpine, when Rv0194 was heterologously expressed in Msm compared with a vector control (Danilchanka, Mailaender and Niederweis 2008). The rapid increase of ethidium upon addition of reserpine suggests that Rv0194 actively exports ethidium bromide. But as acknowledged by the authors of this study, an indirect effect of Rv0194, by exporting lipids that affect membrane permeability and thereby reduce β-lactam influx, cannot be excluded (Danilchanka, Mailaender and Niederweis 2008). Vancomycin and β-lactams act in the periplasm, which raises the question of how an inner membrane protein can confer resistance to these antibiotics. In gram-negative bacteria, export from the periplasm occurs via tripartite efflux pumps, composed of a transporter located in the inner membrane, a periplasmic adaptor protein and an outer membrane channel (Wang *et al*. 2017). Drugs are taken up from the periplasm or cytosol by the inner membrane transporter and expelled to the extracellular space via the continuous channel formed by the tripartite efflux pump. No homologues of the periplasmic and outer membrane components of tripartite efflux pumps have been identified thus far in mycobacteria.

#### Rv2936-2938 (DrrABC)

The ABC transporter DrrABC is composed of the nucleotide binding domain Rv2936 (DrrA) and the membrane domains Rv2937 (DrrB) and Rv2938 (DrrC). It most likely forms a heteromeric complex (DrrA_2_/DrrB/DrrC) and, based on sequence homology of the transmembrane domains, belongs to the type II ABC exporters (Fig. 3) (Rempel, Stanek and Slotboom 2019). It was annotated as a daunorubicin resistance (Drr) transporter due to its sequence similarity to DrrAB of *Streptomyces peucetius* (Cole *et al*. 1998). This bacterium produces doxorubicin and daunorubicin, but is self-resistant to the compounds due to Drr proteins, such as the exporter DrrAB, encoded within the doxorubicin biosynthetic gene cluster (Guilfoile and Hutchinson 1991; Kaur and Russell 1998). In mycobacteria, the DrrABC genes are within the phthiocerol dimycocerosate (PDIM) synthesis and transport transcriptional unit and DrrA, DrrB and DrrC have been implicated in PDIM transport (Camacho *et al*. 2001; Waddell *et al*. 2005; Murry *et al*. 2009). In the extensive genome-wide association study of 6465 Mtb clinical isolates, a high mutation frequency of DrrA in extensively drug-resistant Mtb strains was also observed, suggesting a potential role in drug resistance (Coll *et al*. 2018). DrrA and DrrB, but curiously not the entire DrrABC complex, have been investigated as putative drug efflux pumps. When DrrA was overexpressed in *E. coli*, a 4× increase in the MIC of rifampicin was observed compared with a vector control (Pang *et al*. 2013). Overexpression of DrrA/DrrB in *E. coli* and Msm was correlated with increased MICs of various antibiotics, including the first-line tuberculosis drug ethambutol in Msm. Further, accumulation of [^14^C]doxorubicin was reduced in both heterologous expression systems compared with uninduced control cells and the effect was reversed by addition of reserpine (Choudhuri *et al*. 2002). These experiments suggest active export of drugs by DrrAB, but lack controls such as the inclusion of a mutation that renders the NBD (DrrA) catalytically inactive. In view of the clearly demonstrated role of the DrrABC transporter in the context of PDIMs transport across the inner membrane (Murry *et al*. 2009), the suggested drug efflux capacity of DrrAB remains to be confirmed in Mtb.

#### Rv1634

The MFS transporter Rv1634 was suggested to confer resistance to fluoroquinolones, because Msm expressing the Mtb homologue Rv1634 showed a 2–4-fold increase in the MIC of fluoroquinolones (De Rossi *et al*. 2002). A fluorometric accumulation assay using the heterologous expression system, in which the native homologue MSMEG_3815 had not been deleted, showed that accumulation of norfloxacin was reduced by 25% compared with an Msm vector control (De Rossi *et al*. 2002). It is not possible to distinguish if overexpression of Rv1634 causes increased active drug efflux or decreased drug influx from these data.

#### Rv2333c (Stp)

The MFS transporter Rv2333c was implicated in drug transport by Ramon-Garcia *et al*. (2007). Its function as a putative drug efflux pump was investigated in *M. bovis* in which the Rv2333c homologue was deleted or Rv2333c was expressed from a plasmid. Inactivation of the Rv2333c homologue correlated with 2–4-fold decreased MICs of spectinomycin and tetracycline, and a 25% increase in intracellular [^3^H]tetracycline accumulation compared with the WT. The opposite was observed when Rv2333c was overexpressed—MICs increased by 2-fold and [^3^H]tetracycline accumulation decreased by 25% compared with WT (Ramón-García *et al*. 2007). To unambiguously annotate Rv2333c as a drug efflux pump, the mechanism underlying the changes in tetracycline accumulation, i.e. active drug efflux or changes in the cell wall composition or permeability, remain to be investigated.

#### Other transporters

At least five other transporters have been proposed as drug efflux pumps in Mtb, relying only on data from MIC assays and/or expression studies in clinical strains or under antibiotic stress. To date, very little is known about the MFS transporter Rv0849. It is described as a (putative) drug efflux pump because its deletion caused a reduced MIC of amikacin and two pyrrole compounds (Balganesh *et al*. 2012). The MFS transporter efflux protein A (EfpA, Rv2846c) was named EfpA by Doran *et al*. due to its sequence similarity to other bacterial efflux proteins (Doran *et al*. 1997). Expression levels (Wilson *et al*. 1999; Li *et al*. 2015a,b; Machado *et al*. 2017) and MICs (Li, Zhang and Nikaido 2004) have been analysed but transport assays have not been performed. When the Msm EfpA homologue was deleted, a decreased MIC of fluoroquinolones, but increased MICs of rifampicin, isoniazid, chloramphenicol and erythromycin were observed (Li, Zhang and Nikaido 2004). ABC transporter Rv1218c has been suggested to be a drug transporter alone or in combination with Rv1217c (Balganesh *et al*. 2010; Wang *et al*. 2013). This operon seems to be regulated by TetR-like regulator Rv1219c (Kumar *et al*. 2014). Rv1217c and Rv1218c were upregulated in Mtb multidrug-resistant strains compared with drug-susceptible clinical strains (Wang *et al*. 2013). However, no decrease in MIC values was detected in a Rv1218c KO strain for any drug in clinical use (Balganesh *et al*. 2010). Reduced MIC values for the KO strain were detected for compounds of the pyrrole, pyrazolone and peptidoglycan synthesis inhibitor classes (Balganesh *et al*. 2012; Dinesh, Sharma and Balganesh 2013). Another ABC transporter, Rv1456c-Rv1457c-Rv1458c, has been proposed to be a drug efflux pump because upregulation of its expression has been detected in drug-resistant Mtb strains (Hao *et al*. 2011; Sriraman *et al*. 2018). Similarly, overexpression of Rv2459 (JefA) under rifampicin, isoniazid or ethambutol induced stress has been shown in several studies (Gupta *et al*. 2010a; Li *et al*. 2015b; Narang *et al*. 2017; Ghajavand *et al*. 2019). Overexpression of JefA from a plasmid in Mtb resulted in increased MIC values for isoniazid, ethambutol and streptomycin, while treatment with CCCP or verapamil induced (partial) reversal to WT MICs (Gupta *et al*. 2010b). More data are required to determine the function of these transporters. Increased expression or altered MIC values suggest a potential role as drug efflux pump but are also compatible with functions other than drug efflux. Further investigation of these transporters will increase our understanding of mycobacterial physiology.

### Transporters with other characterized functions

#### Rv2942 (MmpL7)

The *mmpL7* gene is located within the PDIM synthesis and transport transcriptional unit (Cox *et al*. 1999). PDIM export by MmpL7 was demonstrated by comparing the localization of these lipids in a transposon mutant versus WT (Camacho *et al*. 2001). In the same study, disruption of PDIM transport or synthesis affected membrane fluidity and sensitivity to detergents such as sodium dodecyl sulfate. MmpL7 was also proposed to export drugs. Initial evidence for a role in drug resistance came from isolation of isoniazid-resistant Msm strains transformed with Mtb cosmid libraries, one of which contained a cosmid encoding MmpL7 (Pasca *et al*. 2005). The mechanism of resistance was investigated by overexpression of MmpL7 from a plasmid in Msm and monitoring changes in MIC and isoniazid transport compared with a vector control. MmpL7 overexpression increased the MIC of isoniazid and ethionamide by 16- and 4-fold, respectively. The addition of CCCP or reserpine partially restored the MIC of isoniazid. An accumulation assay with radioactively labelled [^14^C]isoniazid showed significantly less accumulation in the Msm strain overexpressing MmpL7. The addition of reserpine led to the rapid accumulation of [^14^C]isoniazid, which was reversed by the addition of glycerol. Although the data were not generated with an inactivated MmpL7 transporter as control, there is considerable evidence that MmpL7 effluxes isoniazid in Msm. However, Msm does not encode a MmpL7 homologue and PDIM is not found in its cell wall (Daffé and Laneelle 1988; Pasca *et al*. 2005). Finally, deletion of the *mmpL7* gene in Mtb did not result in altered susceptibility to isoniazid or ethionamide (Domenech, Reed and Barry 2005), and drug efflux studies in which MmpL7 was overexpressed in Mtb have not been reported. Therefore, the relevance of drug efflux mediated by MmpL7 in Mtb awaits further investigation.

#### Rv1410c (P55)

Rv1410c, or P55, is an MFS transporter encoded in an operon with lipoprotein LprG. P55 was first implicated in mycobacterial drug resistance when the homologue from *M. bovis* BCG was expressed in Msm (Silva *et al*. 2001). P55 KO in *M. bovis* BCG was more sensitive to rifampin, novobiocin, clofazimine and vancomycin (Ramón-García *et al*. 2009). The operon knock-out strain (dKO) in Msm was more susceptible to ethidium bromide. In the same work however, altered colony morphology and defect in sliding mobility was noticed in the dKO strain, hinting at a possible change in cell envelope composition (Farrow and Rubin 2008). The hypothesis that Rv1410c transports lipids across membranes (Ramón-García *et al*. 2009) was supported by the finding that its operon partner lipoprotein LprG is able to bind diacylated and triacylated molecules such as lipoarabinomannan (LAM), lipomannan (LM) and phosphatidylinositol mannosides (PIMs) (Drage *et al*. 2010). Since neither the lipoprotein nor the transporter alone is able to complement the dKO in Msm, *M. abscessus* or Mtb (Farrow and Rubin 2008; Bianco *et al*. 2011; Hohl *et al*. 2019) it is believed that the proteins work in concert in the transport process of their substrate(s). In 2014, two groups independently showed that LprG affects the surface expression of LAMs, which is essential for infection of the host by Mtb (Gaur *et al*. 2014; Shukla *et al*. 2014). However, Martinot and colleagues proposed another mechanism by which Rv1410c and LprG influence infection outcome. By lipidomic analysis of the dKO and operon overexpression strains in Mtb, they showed that Rv1410c and LprG transport triacylglycerides (TAGs). *In vitro* LprG transport assays in lipid vesicles with fluorescently labelled TAG confirmed this finding and the authors hypothesized that the virulence defect of Mtb dKO is due to a defect in mycobacterial metabolism caused by intracellular TAG accumulation (Martinot *et al*. 2016). Finally, transport assays in intact cells comparing WT and dKO Msm strains showed that the accumulation of a fluorescent substrate BCECF did not change in energized and de-energized cells (with CCCP), in both conditions the double KO strain accumulated considerably more BCECF than WT strain (Hohl *et al*. 2019). Together, these results suggest that in the absence of this operon, the cell envelope of mycobacteria becomes compromised and allows for increased influx of some drugs and compounds. In conclusion, Rv1410c is a membrane transporter that exports cell envelope components from the cytoplasm, thus ensuring reduced permeability of Mtb to drugs.

#### Rv1819c (BacA)

Rv1819c is a homodimeric ABC transporter with an exporter fold proposed to import unrelated hydrophilic compounds including the antimicrobial peptide bleomycin and vitamin B_12_ (Domenech *et al*. 2009; Gopinath *et al*. 2013; Rempel *et al*. 2020). Domenech *et al*. initially showed that the Mtb H37Rv *rv1819c* KO strain becomes 16–32-fold more resistant towards bleomycin than WT Mtb, hence showing that Rv1819c in fact influxes this antimicrobial glycopeptide (Domenech *et al*. 2009). Additionally, overexpression of Rv1819c in *E. coli* increased susceptibility to bleomycin (Domenech *et al*. 2009). In a seminal study using transposon screening, Rv1819c was identified as a vitamin B_12_ importer (Gopinath *et al*. 2013). Functional assays in which WT and ATPase deficient Rv1819c were expressed in the *E. coli* ΔFEC strain, which is unable to import vitamin B_12_, provided convincing evidence that Rv1819c imports vitamin B_12_ as well as bleomycin in an ATPase-dependent fashion (Rempel *et al*. 2020). Structural elucidation of Rv1819c revealed an unusually large cavity with a negatively charged surface. Together with biochemical experiments, it was proposed that Rv1819c imports unrelated hydrophilic compounds in a rather nonspecific manner, as no high-affinity binding sites could be identified for vitamin B_12_ or bleomycin in its large translocation cavity (Rempel *et al*. 2020). Nevertheless, negatively charged hydrophilic compounds such as biotin are not imported, presumably due to electrostatic repulsion by the negatively charged cavity surface, providing evidence for some substrate specificity of Rv1819c.

Despite the lack of evidence supporting the role of Rv1819c as a drug efflux pump (Domenech *et al*. 2009; Gopinath *et al*. 2013), its assumed efflux pump nature constitutes the starting hypothesis of many studies (Gupta et al. 2010a; Garima *et al*. 2015; Li *et al*. 2015a; Kanji *et al*. 2017; Machado *et al*. 2018; Ghajavand *et al*. 2019). In these studies, SNPs or the expression levels of ‘putative efflux pumps’ in various drug susceptible and resistant Mtb strains were investigated. Upregulation of Rv1819c was observed for some strains upon treatment with rifampicin and isoniazid (Gupta et al. 2010a; Garima *et al*. 2015; Li *et al*. 2015a; Ghajavand *et al*. 2019). However, such upregulation does not provide sufficient evidence to describe Rv1819c as a drug efflux pump.

#### Rv2477c

Rv2477c is part of the ABC-F subfamily, a family of translation factors that bind to the E-site of the ribosome and modulate translation (Daniel *et al*. 2018; Fostier *et al*. 2021). ABC-F proteins are soluble, cytosolic proteins that contain two nucleotide binding domains, but no transmembrane domains (Fostier *et al*. 2021).

A role of Rv2477c in antibiotic resistance was suggested based on the upregulation of *rv2477c* upon exposure of Mtb to ofloxacin (Gupta *et al*. 2010a), and an SNP in *rv2477c* in an MDR Mtb strain that was associated with kanamycin and amikacin resistance (Faksri *et al*. 2016). Due to its homology to other ABC-F translational factors, the antibiotic resistance linked to Rv2447c may be due to an effect on translation but this hypothesis remains open for investigation. Rv2477c is a cytosolic protein and cannot actively export substrates on its own, nevertheless it has been described as an ‘efflux pump’ (Gupta *et al*. 2010a).

#### Rv0342 and Rv0933

IniA (Rv0342), a predicted dynamin-like protein (Wang *et al*. 2019), and PstB (Rv0933), the nucleotide binding domain of the ABC phosphate transporter (Pst), were suggested to function as an accessory protein to a drug efflux pump (Colangeli *et al*. 2005) and a drug efflux pump (Banerjee *et al*. 1998; Bhatt, Banerjee and Chakraborti 2000; Gupta *et al*. 2006; Oh *et al*. 2017), respectively. This was based on reduced tolerance of the Mtb H37Rv *iniA* gene deletion strain to isoniazid and ethambutol (Colangeli *et al*. 2005), increased MIC of fluoroquinolones against an Msm strain overexpressing PstB (Banerjee *et al*. 2000) and reduced tolerance to fluoroquinolones in a *pstB* deletion strain (Bhatt, Banerjee and Chakraborti 2000). A GTP dependent fission of membranes by IniA was demonstrated by extensive *in vitro* characterization and structure determination (Wang *et al*. 2019) and phosphate import by the mycobacterial Pst transporters (Braibant *et al*. 1996; Banerjee *et al*. 1998; Bhatt, Banerjee and Chakraborti 2000) was also observed *in vitro*. No other experimental data are available to conclude that these proteins do or do not export drugs.

## CONCLUSIONS AND RECOMMENDATIONS

In this review, we summarize ambiguities associated with the study and clinical role of drug efflux pumps.

The problem has many facets. Many transporters have been annotated as drug efflux pumps based on homology to other already characterized or proposed drug efflux pumps. Such annotations can be self-fulfilling prophecies. If one sets out to study the function of a membrane transporter annotated as drug efflux pump, the straightforward experiment is to generate a gene deletion or establish membrane protein overexpression to determine whether the MIC of different drugs and dyes changes. This creates almost by definition a positive bias in two ways: (i) if a drug efflux phenotype is suspected, often it is just a matter of finding the right compound or the right experimental condition and (ii) reporting positive results (namely Rv-XY is a drug efflux pump) in a publication has a higher impact and probability of success than negative results (namely that a transporter of interest is not an efflux pump).

The next challenge is the generation and interpretation of data to characterize a drug efflux pump. As we highlight in this review, it is common praxis in the field to (i) use expression levels of suggested drug efflux pumps as a measure for drug efflux, which is the most misleading approach in our view, (ii) compare overexpressed or complemented transporter versus empty vector instead of using an inactivating mutation and (iii) include efflux inhibitors (which are often cationic amphiphiles and/or have the propensity to nonspecifically insert into phospholipid bilayers) during MIC determination experiments, thereby interfering with a large number of active membrane transport processes and cell wall functions such as the PMF and all processes that depend on it over a prolonged time period.

For many putative drug efflux pumps discussed in this review, while available data clearly suggest a role in drug resistance, they are insufficient to conclude the underlying mechanism. In contrast, available data strongly support active export of drugs or dye complexes by MmpL5, Tap, Mmr and Rv2686c-2688c. Yet, with the exception of the role of MmpL5 in conferring resistance to bedaquiline, direct causal relationships between drug efflux pumps (and corresponding mutations either in the drug efflux pump gene or its regulatory proteins) and antitubercular drug resistance are essentially lacking. Besides, no drug efflux pumps that play a dominant role in multidrug efflux in Mtb, such as the tripartite RND systems AcrAB-TolC in *E. coli* or MexAB-OprM in *P. aeruginosa*, have been characterized.

A growing body of literature finds and assigns novel roles to membrane transporters that were initially characterized as drug efflux pumps. Examples are Rv1410c (involved in transport of triacylated lipids), DrrABC and MmpL7 (transport of PDIMs) or Rv1819c (import of vitamin B_12_) to name the most prominent ones. It is therefore conceivable, and it can be hoped for that more such re-annotations will be reported in the coming years and decades. It is plausible that many of these transporters play important roles in the overall physiology of Mtb, which might include the enigmatic tolerance to drugs and the difficulty to sterilize Mtb infections. However, they likely do not do so via direct drug efflux, but via indirect processes, such as the biosynthesis of the highly complex and formidable mycobacterial cell envelope, or by transporting metabolites (both toxic and essential ones) across the inner membrane.

Perhaps the future of mycobacterial transporter and efflux pump elucidation lies in the recent spectacular advances of structural biology. Of the mycobacterial transporters discussed in this review, structures are only available for Rv1819 and IniA (a dynamin like protein and not a transporter). With cryo-electron microscopy becoming firmly established as a technique for high-resolution structure determination, more structures of putative mycobacterial drug efflux pumps might bring surprises, such as for Rv1819c, which has an ABC exporter fold but was shown to have an importer function, raising interesting questions about the mechanism of transport.

We hope that this review paves the way for a more rigorous genetic, biochemical and structural characterization of membrane transporters classified as drug efflux pumps in Mtb and beyond.

## Supplementary Material

fuab050_Supplemental_File
