# Supplementary material for: Critical discussion on drug efflux in Mycobacterium tuberculosis
Source: FEMS Microbiol Rev. 2021 Oct 12;46(1):fuab050. doi: 10.1093/femsre/fuab050 (PMC8829022; doi:10.1093/femsre/fuab050)
Supplement: fuab050_Supplemental_File [file fuab050_supplemental_file.docx]

Table 1: Overview of the data available on putative mycobacterial drug efflux pumps.

* Based on homology models generated using the SwissModel server.

**Studies in which the upregulation, expression levels or SNPs of putative drug efflux pumps were investigated are not included.
